# Survival trends and prognostic factors in patients with solitary plasmacytoma of bone: A population‐based study

**DOI:** 10.1002/cam4.3533

**Published:** 2020-11-03

**Authors:** Xuxing Shen, Shu Liu, Chao Wu, Jing Wang, Jianyong Li, Lijuan Chen

**Affiliations:** ^1^ Department of Hematology the First Affiliated Hospital of Nanjing Medical University Jiangsu Province Hospital Nanjing China; ^2^ Department of radiation oncology the First Affiliated Hospital of Nanjing Medical University Jiangsu Province Hospital Nanjing China

**Keywords:** prognostic factor, solitary plasmacytoma of bone, survival trends

## Abstract

Solitary plasmacytoma of bone (SPB) is a single, isolated plasmacytoma originated from the bone. The survival trends of patients with SPB in recent years remain unknown. And the prognostic system of SPB may also need to be refined. The 18 Surveillance, Epidemiology, and End Results (SEER) databases of the National Cancer Institute in the United States were used to extract data for this study. The third edition of the International Classification of Disease for Oncology (ICD‐O‐3) code 9731 was used to identify cases of SPB. For each case, factors including age at the time of diagnosis, sex, race, marital status, insurance status, primary sites of tumors, and the use of surgery were collected. The outcomes of patients with SPB were compared between two groups. And the prognostic impacts of baseline characteristics and use of surgery was studied. A total of 4103 (from 1976 to 2016) cases of SPB were identified. The median age was 65 years old. Patients in time period‐2 (2008–2016) show better survival as compared to those in time period‐1(1976–2007) (median overall survival: 88 months *vs*. 73 months, *p* = 0.0332). Age ≤ 65 years and being male were associated with better outcomes. The widowed individuals had significantly inferior survival and myeloma‐specific survival than the single, married, or divorced individuals (*p* values all <0.0001). Patients with lesions in bones of skull and face and associated joints had longer survival as compared with those with bone lesions in other sites (median overall survival: 107 months *vs*. 79 months, *p* = 0.0694). The use of surgery was significantly associated with improved survival (median survival: surgery performed 98 months *vs*. not performed 73 months, hazards ratio [HR]: 0.7623, 95% CI: 0.7009–0.8472; *p* < 0.0001) and myeloma‐specific survival (median myeloma‐specific survival: surgery 160.0 months *vs*. no surgery 143.0 months, HR: 0.8469, 95% CI: 0.7493–0.9572; *p* = 0.0078). Multivariable analysis revealed that surgery, marital status, and age were independent prognostic factors for overall survival in patients with SPB. The improvement in the survival of patients with SPB has been observed in recent years. And several potential prognostic factors were identified. Future prospective studies are warranted to explore the roles of novel agents, surgery, and systemic chemotherapy in the treatment of SPB.

## Graphical abstract

This study used SEER databases to analyze the prognostic system of solitary plasmacytoma of bone. The results showed that age ≤65 years and being male were associated with better outcomes. The widowed individuals had significantly inferior survival than the others. In addition, the use of surgery was significantly associated with improved survival.

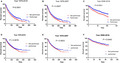

## 1 INTRODUCTION

Plasma cell neoplasms, which include plasma cell myeloma (PCM), plasmacytoma, and others, are caused by the clonal expansion of immunoglobulin secreting, heavy chain class‐switched, and terminally differentiated B cells. Solitary plasmacytoma refers to a single localized tumor consisting of monoclonal plasma cells without any clinical evidence of PCM or physical or radiographical evidence of additional plasma cell neoplasms. Solitary plasmacytoma is relatively rare, accounting for only 3% of all plasma cell neoplasms. Based on the site of lesion, solitary plasmacytoma is classified into solitary plasmacytoma of bone (SPB) and solitary extramedullary plasmacytoma (SEP).^1^ SPB is a single, isolated plasmacytoma originated from the bone,^2^, ^3^ while solitary extramedullary plasmacytoma is characterized by the presence of plasma cells infiltrating in soft tissue mass.^4^, ^5^

SPB accounts for 1–2% of plasma cell tumors. It shows male predominance, with 65% of patients being male. The median age at disease onset is approximately 55 years. Bones with active hematopoiesis are the most common sites involved by SPB. The most frequently affected bone is the vertebrae, which is followed by ribs, skull, and pelvic bones. The standard therapy for SPB is radiotherapy, even in patients in whom the tumor is completely resected. Despite the high local control rate with radiotherapy, the rate of recurrence of SPB is high, with approximately two‐thirds of cases eventually progress to PCM or additional solitary or multiple plasmacytomas. Therefore, it is important to find robust prognostic factors to identify patients who are at higher risk of relapse to use risk‐adapted therapy for treating patients with SPB. The risk factors for progression include older age, tumor size > 5 cm, and persistence of M protein for >1 year following radiation. However, the findings come from small respective series.^6^, ^7^ Therefore, prospective studies or large retrospective studies are needed to confirm the prognostic roles of the risk factors or explore novel prognostic factors.

The advent of novel agents has substantially changed the therapeutic landscape of PCM and improved the outcomes of patients with PCM. Some population‐based studies have confirmed that the survival of PCM patients has significantly improved in recent years.^8^, ^9^, ^10^ In contrast to patients with PCM, the therapeutic roles of novel agents in the SPB treatment remain unknown. And the survival trends of patients with SPB are yet to be defined.

We hypothesized that the survival of patients with SPB has improved with the introduction of novel agents. Therefore, we used the Surveillance, Epidemiology, and End Results (SEER) program to study the survival trends of patients of SPB. Additionally, using the SEER program, we also investigated the prognostic roles of some baseline characteristics to identify some potential prognostic factors.

## 2 MATERIAL AND METHODS

### 2.1 Data source

The 18 SEER databases of the National Cancer Institute in the United States were used to extract data for this study. SEER is a program that collects and publishes cancer incidence, treatment, and survival data from population‐based cancer registries, representing approximately 28% of the US population. The 18 SEER registries including Atlanta, Detroit, Greater California, Greater Georgia, Hawaii, Iowa, Kentucky, Los Angeles, New Mexico, New Jersey, Rural Georgia, states of Connecticut, San Francisco‐Oakland, Seattle‐Puget Sound, San Jose‐Monterey, the Alaska Native Tumor Registry, Louisiana, and Utah were used to conduct this study.

### 2.2 Data collection

The third edition of the International Classification of Disease for Oncology (ICD‐O‐3) code 9731 was used to identify cases of SPB. Cases with the involvement of bone marrow, peripheral blood, or other extramedullary organs were not considered as SPB, and therefore excluded from the present study. For each case, sociodemographic factors including age at the time of diagnosis (4–97 years old), sex (male or female), race (black, white or other), marital status (married, unmarried, divorced, separated, single or widowed) and insurance status (Medicaid, insured, or uninsured) were collected. Primary sites of tumors were labeled in detail. We further classified bones of the skull, face and associated joints and mandible as bones of the head, face, and jaws; long and short bones of the upper limb and associated joints as bones of the upper limb; long and short bones of the lower limb and associated joints as bones of the lower limb; rib, sternum, clavicle, and associated joints as bones of the chest; pelvic bones, sacrum, coccyx, and associated joints as bones of the pelvis. Cases from these sites above and the vertebral column were included to study the impacts of sites of lesions on the survival of patients with SPB.

### 2.3 Statistical analysis

Categorical variables were compared between the two groups using Fisher's exact test or the chi‐square test. Overall survival (OS) was defined as time from diagnosis to death or last follow‐up. Myeloma‐specific survival was defined as time from diagnosis to death from myeloma or last follow‐up. Survival curves were plotted using the Kaplan‐Meier method. The log‐rank test was used for comparing the difference in survival. *p*‐value was 2‐sided and *p* < 0.05 was considered to be statistically significant. All analyses were conducted using GraphPad Prism 5 statistical software.

## 3 RESULTS

### 3.1 Patients

A total of 4103 (from 1976 to 2016) cases of SPB were identified. The median age was 65 years old (IQR: 55–74 years). Patients with SPB showed male predominance (61.6%). Other baseline characteristics were summarized in Table 1. Median follow‐up time was 41 months (0–416 months).

**TABLE 1:** The baseline characteristics of patients with solitary plasmacytoma of bone.

Baseline characteristics	Total cases, n = 4103
**SEER registry**	
Alaska Natives	9 (0.2%)
Atlanta	216 (5.3%)
California	853 (20.8%)
Connecticut	156 (3.8%)
Detroit	305 (7.4%)
Greater Georgia	311 (7.6%)
Hawaii	65 (1.6%)
Lowa	247 (6.0%)
Kentucky	283 (6.9%)
Los Angeles	476 (11.6%)
Louisiana	239 (5.8%)
New Jersey	428 (10.4%)
New Mexico	106 (2.6%)
Rural Georgia	9 (0.2%)
San Francisco‐Oakland SMSA	218 (5.3%)
San Jose‐Monterey	92 (2.2%)
Seattle	326 (7.9%)
Utah	109 (2.6%)
**Median age**	63 years
**Male sex**	2526 (61.6%)
**Race**	
White	3310 (80.7%)
Black	591 (14.4%)
Asian or Pacific Islander	150 (3.7%)
American Indian/Alaska Native	33 (0.8%)
**Marital status**	
Single (Unmarried + separated)	560 (13.6%)
Married	2516 (61.3%)
Divorced	335 (8.2%)
Widowed	483 (11.8%)
**Surgery**	
Not performed	3002 (73.2%)
Performed	1076 (26.2%)
**Insurance status**	
Not insured	363 (8.8%)
Insured	2005 (48.9%)

### 3.2 Survival trends of patients with solitary plasmacytoma

**FIGURE 1:**
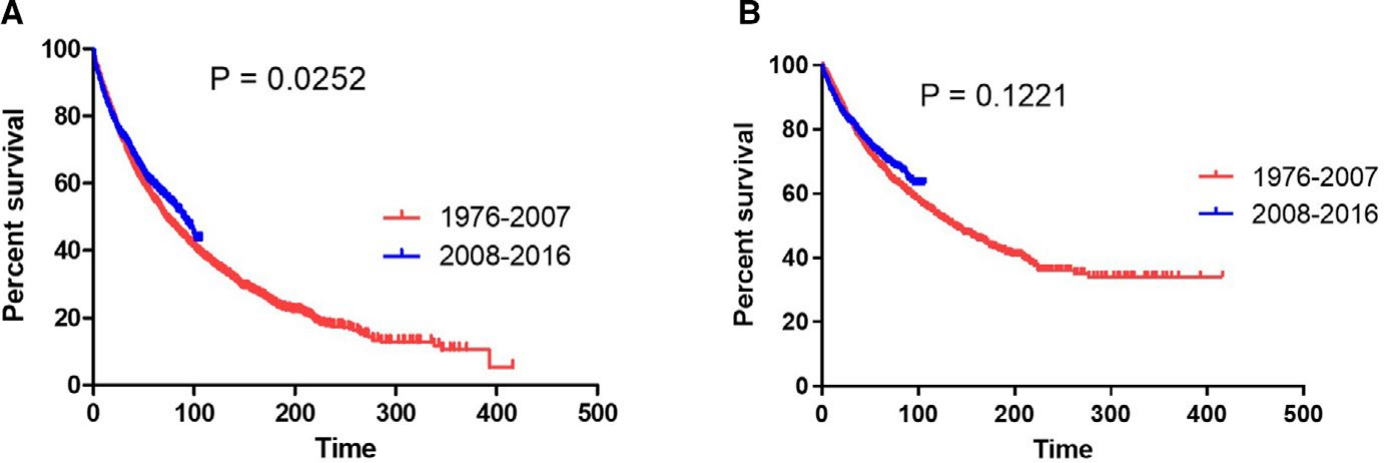
Overall survival (A) and myeloma‐specific survival (B) of patients with solitary plasmacytoma of bone in different time periods

We classified these patients into two groups according to the year of diagnosis: 1976–2007 and 2008–2016. Two thousand and three hundred and eight patients and 1795 patients were diagnosed during 1976–2007 (time period‐1) and 2008–2016 (time period‐2), respectively. The baseline characteristics of patients were compared between time period‐1 and time period‐2 (Table 2). We then compared the outcomes of patients with solitary plasmacytoma between time period‐1 and time period‐2. We found that patients in time period‐2 show better survival as compared to those in time period‐1 (median OS: 88 months *vs*. 73 months, *p* = 0.0252; Figure 1A). The 100‐month survival rates for patients diagnosed in time period‐1 and time period‐2 were 57.6% and 63.1%, although the difference was not statistically significant (*p* = 0.1221, Figure 1B).

**TABLE 2:** Differences in the baseline characteristics between patients with solitary plasmacytoma of bone from two eras.

	1976–2007 (n = 1795)	2008–2016(n = 2308)	*p* value
Sex (no.%)			
Female	674 (37.5%)	903 (39.1%)	0.3160
Male	1121 (62.5%)	1405 (60.9%)	
Age (no.%)			
≤65	696 (38.8%)	890 (38.6%)	0.8972
>65	1099 (61.2%)	1418 (61.4%)	
Race (no.%)			
White	1489 (83.2%)	1821 (79.4%)	0.0065
Black	234 (13.1%)	357 (15.6%)	
Asian or Pacific Islander	51 (2.8%)	99 (4.3%)	
American Indian/Alaska Native	16 (0.9%)	17 (0.7%)	
Insurance (no.%)			
Yes	129 (84.9%)	1875 (84.7%)	>0.9999
No	23 (15.1%)	339 (15.3%)	
Surgery (no.%)			
Performed	531 (29.8%)	545 (23.7%)	<0.0001
Not performed	1251 (70.2%)	1750 (76.3%)	
Marital (no.%)			
Single	219 (12.6%)	337 (15.7%)	0.0072
Married	1136 (65.5%)	1379 (64.0%)	
Divorced	141 (8.1%)	193 (9.0%)	
Widowed	239 (13.8%)	243 (11.3%)	

### 3.3 The impacts of demographic characteristics on the outcomes of patients

**FIGURE 2:**
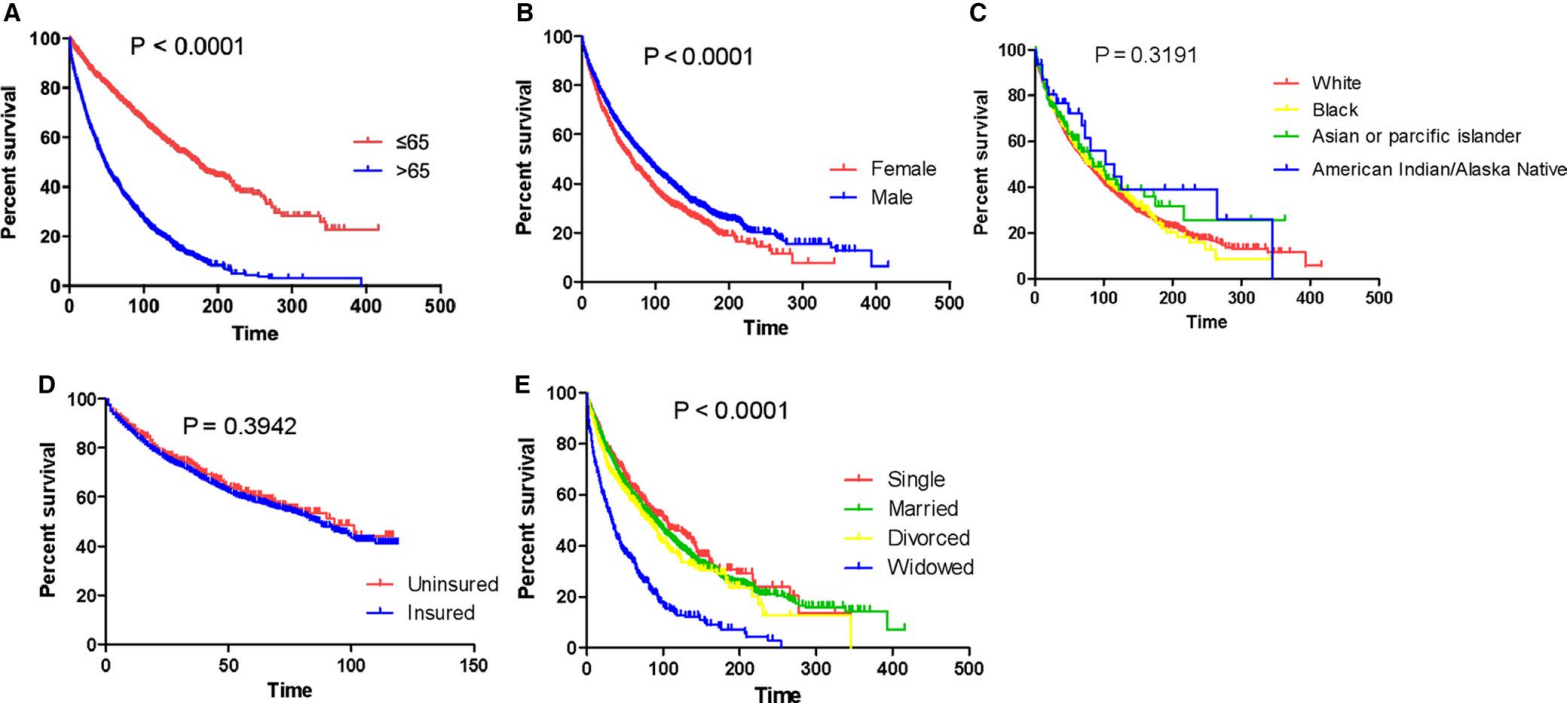
Survival of patients with solitary plasmacytoma of bone stratified by age (A), sex (B), race (C), insurance (D), and marital status (E)

The prognostic impacts of demographic characteristics including age, gender, races, marital status, and insurance status were analyzed in the present cohort. Age ≤ 65 years significantly predicted better OS (HR: 0.3407, 95% CI: 0.3115–0.3726; *p* < 0.0001, Figure 2A) and myeloma‐specific survival (HR: 0.3342, 95% CI: 0.2966–0.3765; *p* < 0.0001, supplemental Figure S1A) in patients with SPB. Male patients had significantly longer survival (HR: 0.8147, 95% CI: 0.7372–0.8847; *p* < 0.0001, Figure 2B) and myeloma‐specific survival (HR: 0.7052, 95% CI: 0.6271–0.7931; *p* < 0.0001, supplemental Figure S1B) than female patients. Patients of different ethnic groups showed similar survival (*p* = 0.3191, Figure 2C) and myeloma‐specific survival (*p* = 0.3623, supplemental Figure S1C). A total of 3891 cased were included to analyze the impact of the marital status on OS. Regarding the marital status, 2516, 560, 335, and 483 patients were married, single, divorced, and widowed respectively. The 10‐year OS rates for single, married, divorced, and widowed patients were 46.1%, 40.1%, 36.1%, and 12.7%. The widowed individuals had significantly inferior survival and myeloma‐specific survival than the single, married, or divorced individuals (*p* values all <0.0001, Figure 2D, supplemental Figure S1D). A total of 2366 cases were available for analyzing the effects of insurance status on OS. Insured individuals had similar survival (insured 86 months *vs*. uninsured 93 months, *p* = 0.3942, Figure 2E) and myeloma‐specific survival (insured not reached *vs*. uninsured not reached, *p* = 0.2259, supplemental Figure S1E) to those uninsured patients or Medicaid patients.

### 3.4 The effects of sites of lesions on patient outcomes

**FIGURE 3:**
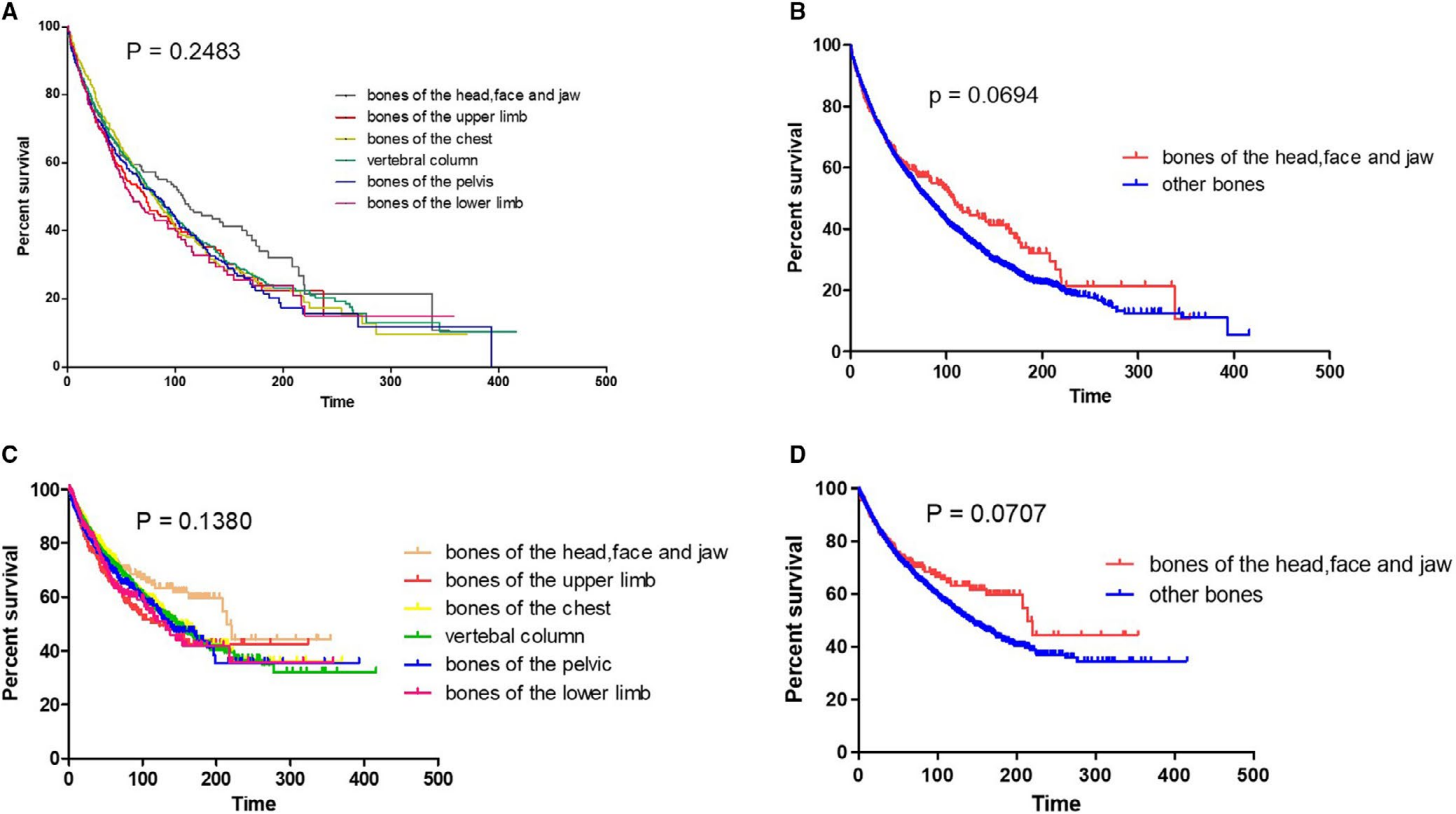
Overall survival (A) and myeloma‐specific survival (C) of patients with solitary plasmacytoma of bone (SPB) according to different sites of lesion. Overall survival (B) and myeloma‐specific survival (D) of patients with SPB from bones of skull, face, and jaw and those from other sites

The specified information regarding locations of lesions was available for 3839 patients. Among these patients, the most frequently involved site was the vertebral column (1670, 43.5%), which was followed by bones of the pelvis (631, 16.4%) and bones of the chest (605, 15.8%). The sites of bone lesions had no significant impacts on the survival (*p* = 0.2483, Figure 3A) and myeloma‐specific survival (*p* = 0.1380, Figure 3C) of patients. However, we found that patients with lesions in bones of skull and face and associated joints had longer survival (107 months *vs*. 79 months, *p* = 0.0694, Figure 3B) and myeloma‐specific survival (214 months *vs*. 141 months, *p* = 0.0707, Figure 3D) as compared with those bone lesions in other sites, although the difference was not statistically significant.

### 3.5 Surgery and outcomes

**FIGURE 4:**
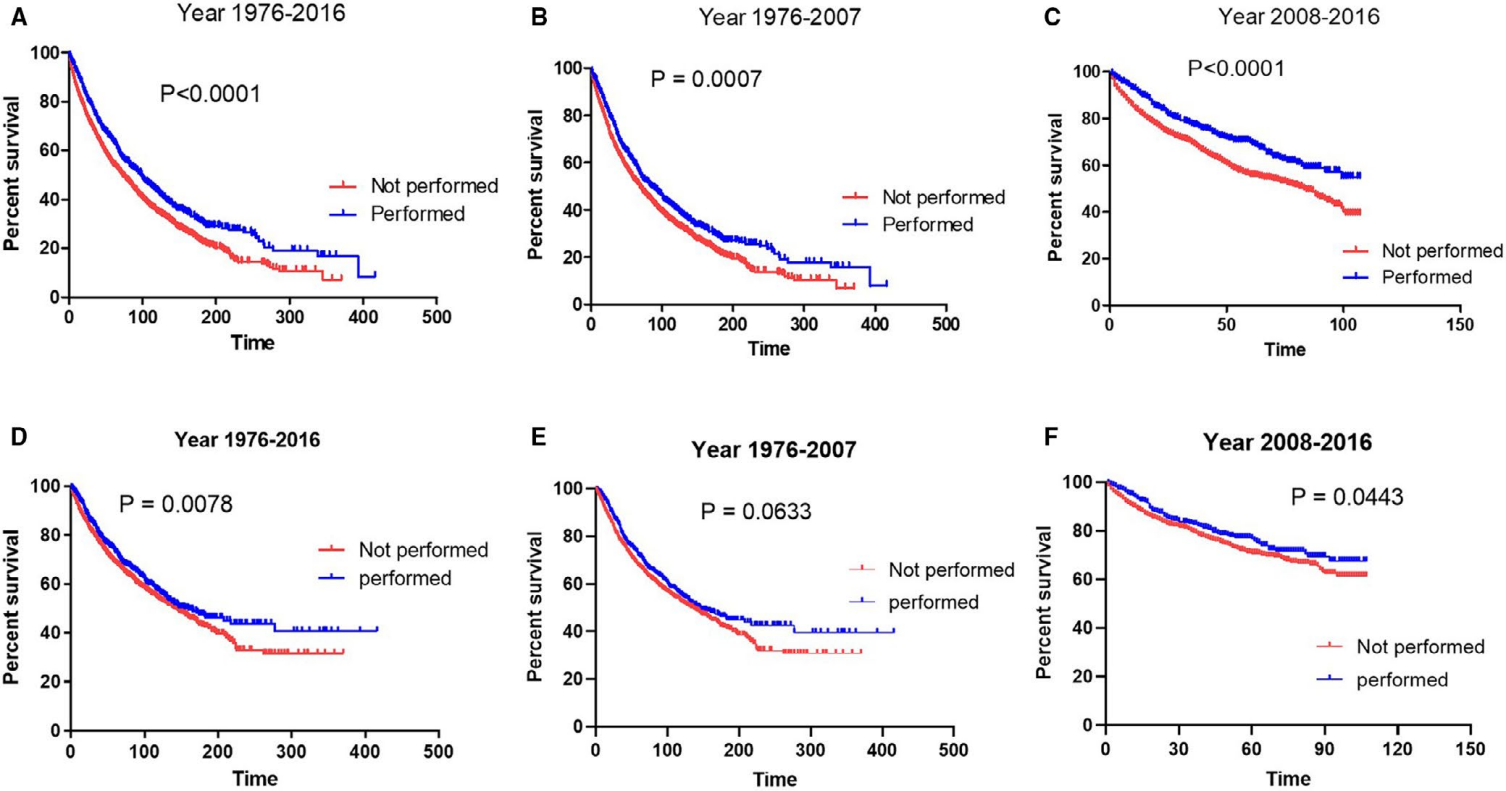
The impacts of surgery on the overall survival (A) and myeloma‐specific survival (D) of patients with solitary plasmacytoma of bone (SPB). The impacts of surgery on the overall survival and myeloma‐specific survival of SPB patients diagnosed from 1976–2007 (B, E) and those diagnosed from 2008–2016 (C, F)

We then analyzed the impact of surgery on survival in patients with SPB. We found that the use of surgery was significantly associated with improved survival (median survival: surgery 98 months *vs*. no surgery 73 months, HR: 0.7623, 95% CI: 0.7009–0.8472; *p* < 0.0001, Figure 4A) and myeloma‐specific survival (median myeloma‐specific survival: surgery 160.0 months *vs*. no surgery 143.0 months, HR: 0.8469, 95% CI: 0.7493–0.9572; *p* = 0.0078, Figure 4D). We separately analyzed the prognostic role of surgery in patients diagnosed in time period‐1 and time period‐2. We found that, in patients diagnosed in time period‐1, patients who had surgery had significantly longer survival than those who did not have surgery (median survival: surgery 84 months *vs*. no surgery 68 months; HR: 0.8106, 95% CI: 0.7246–0.9158; *p* = 0.0007, Figure 4B). The survival benefit of surgery was also confirmed in patients with SPB diagnosed in time period‐2 and this survival benefit was more remarkable that in time period‐1 (median survival: surgery not reached *vs*. no surgery 83 months; HR: 0.6606, 95% CI: 0.5822–0.8055, *p* < 0.0001, Figure 4C). Use of surgery was marginally associated with improved myeloma‐specific survival (*p* = 0.0633, Figure 4E) in time period‐2 and significantly associated with improved myeloma‐specific survival (*p* = 0.0433, Figure 4F). In patients with SPB in vertebral column, surgery was also significantly associated with improved survival (median survival: surgery 96 months *vs*. no surgery 77 months; HR: 0.6606, 95% CI: 0.5822–0.8055, *p* = 0.0005, supplemental Figure S2).

### 3.6 Multivariable analysis of prognostic factors in predicting overall survival for patients with spb

We further included factors including time period, gender, use of surgery, marital status, and age in multivariable analysis of survival for patients with SPB. We found that surgery, marital status, and age were independent prognostic factors for overall survival in patients with SPB (Table 3).

**TABLE 3:** Multivariable analysis of prognostic factors for overall survival for patients with solitary plasmacytoma of bone.

	Multivariate analysis	
	HR (95% CI)	*p* value
Year (1976–2007 *vs* 2008–2016)	1.095 (0.994–1.207)	0.065
Gender (Female *vs* male)	1.028 (0.936–1.130)	0.561
Surgery (not performed *vs* performed)	1.164 (1.052–1.287)	0.003
Marital status (Widowed *vs* others)	1.459 (1.288–1.654)	<0.001
Age (>65 *vs* ≤65)	2.914 (2.645–3.209)	<0.001

Abbreviations: CI, confidence interval; HR, Hazard ratio.

## 4 DISCUSSION

Solitary bone plasmacytoma is a rare, malignant plasma cell disorder with a relatively high incidence of recurrence.^11^, ^12^ Owing to the rarity of this disease, there is a paucity of data on survival trends and prognosis factors of this disease. Here, we used the SEER database to evaluate survival trends and prognostic factors of patients with solitary bone plasmacytoma.

In this study, by comparing the survival of patients from two eras, we found that the survival of patients with solitary plasmacytoma improved in the last decade (median survival: 88 months *vs*. 73 months, *p* = 0.0332). The improvement in the survival of patients diagnosed during 2008–2016 could be possibly attributed to the use of novel agents, which include proteasome inhibitors and immunomodulatory drugs. The role of novel agents has not been defined in the treatment of patients with solitary plasmacytoma, however, the novel agents could be effective in improving the survival of patients who progressed to multiple myeloma, since the efficacy of the novel agents in treating relapsed/refractory multiple myeloma.^7^, ^13^ The use of bortezomib in treating solitary plasmacytoma has been reported in several case reports, which suggested the possible efficacy of bortezomib in treating solitary plasmacytoma. Additionally, in a single‐center retrospective study by Mignot et al., patients with solitary plasmacytoma treated with lenalidomide‐dexamethasone with intensity‐modulated radiation therapy (IMRT) had longer multiple myeloma‐free survival and progression‐free survival than treated IMRT alone.^14^ In this study, the majority of patients had SPB (87%), suggesting the addition of lenalidomide to radiotherapy could improve the outcomes of patients with SPB. However, further prospective clinical trials are needed to define the therapeutic role of lenalidomide in patients with SPB. However, in our study, it should be noted that the improvement in overall survival has not been corrected for population life expectancy. The possibility that the improvement in survival of patients with SPB is caused by the improvement in survival of general population could not be excluded.

Several prognostic factors were identified in this study. Our study showed the prognostic effect of the younger age, which was consistent with the findings of previous studies.^15^, ^16^ We found that male patients with SPB had better survival than female patients. According to the study by Ramsingh et al., being female was independently associated with worse disease‐specific survival in patients with solitary plasmacytoma.^17^ The reasons for the negative prognostic impact of the female gender remain to be determined. According to the study by Ramsingh et al., black people with solitary plasmacytoma showed significantly worse disease‐specific survival and OS than those of other races. In contrast, the race did not show significant prognostic effects in patients with SPB in our study. Different populations included in these two studies might account for the discordance. In our study, we only included patients with SPB, while the study by Ramsingh *et al* included all the patients with solitary plasmacytoma. Additionally, the study by Ramsingh et al. only included patients diagnosed from 1988 to 2004, while our study included patients diagnosed from 1976 to 2016.

The prognostic impacts of sites of lesions in patients with SPB remain unknown. And the prognostic value of spinal involvement remains controversial.^7^ In the present study, patients with SPB arising from the spine had similar survival to patients with SPB from other sites. Interestingly, we found patients with SPB in bones of skull and face had better survival, although the survival benefit was not statistically significant. The reasons for this phenomenon remain unknown, one possible explanation is that SPB in bones of skull and face may always lead to an early presentation, thereby contributing to a better outcome. Our study suggests site information may improve the risk of stratification of patients with SPB.

According to the guidelines for the treatment of SPB, radiation remains the mainstay of the treatment.^1^, ^18^, ^19^ And radiotherapy provides long‐term local control in patients with SPB.^18^ Surgery without radiotherapy was associated with inferior outcomes in patients with SPB.^20^ The added benefit of surgery to radiation in patients with SPB remains controversial. Some previous studies suggest that radiotherapy combined with surgery was associated with better outcomes in patients with SPB.^20^, ^21^ In our analysis, we found that patients who were treated with surgery had better survival and myeloma‐specific survival than those who did not receive surgery, suggesting surgery might improve the prognosis of patients with SPB. In patients with SPB in vertebral column, we found that use of surgery was associated with improved survival. The reason for the improvement in survival caused by surgery is not well defined. However, we postulate that surgery helps reduce the tumor burden and may eradicate the clones that are potentially resistant to radiation therapy, thereby contributing the improvement in survival. And the survival benefit associated with surgery was more remarkable in time period‐2, indicating the role of surgery had been improved in recent years. This could be attributed to the tremendous progress in the imaging SPB.^22^ The use of whole‐body positron emission tomography‐computed tomography (PET/CT) or magnetic resonance imaging (MRI) enables precise staging of SPB, which further allows the accurate and complete resection of the tumor.^23^, ^24^ In the study by Wang et al, radiotherapy combined with surgery was significantly associated with improved survival in patients with SPB in vertebral column, and in their study, most patients who received surgery were also treated with radiation. Therefore, the prognostic effect of surgery in patients with SPB in vertebral column could be attributed to its association with combination of surgery and radiation. Additionally, patients who received surgery and radiation had significantly better survival than those treated with radiation alone, highlighting the importance of use of surgery.

Although a similar analysis was performed in the study by Goyal et al, our study is different to theirs in several aspects. First, the study by Goyal et al used the National Cancer Database, while we used the SEER database. Second, we studied patients diagnosed from 1976 to 2016, while they only included patients from 2000 to 2011. Third, we also studied the prognostic factors including marital and insurance status in patients with SPB. Moreover, they also included cases with extraosseous involvement, while we only included cases with osseous involvement. However, this study has some limitations. First, despite the large sample size, the current study is retrospective, making the conclusions less definitive and robust. The detailed information regarding the use of radiation, novel agents, or systemic chemotherapy was not available for analyzing the impacts of these treatments on the survival of patients with SPB.

In conclusion, using the SEER database, we analyzed the survival trends and prognostic factors for patients with SPB. The improvement in the survival of patients with SPB has been observed in recent years. And several potential prognostic factors were identified. Future prospective studies are warranted to explore the roles of novel agents, surgery, and systemic chemotherapy in the treatment of SPB.

## CONFLICT OF INTEREST

The authors declare no conflict of interest.

## AUTHOR CONTRIBUTIONS

XXS wrote the manuscript and designed the tables. SL designed the figures. CW revised the manuscript. JW contributed to the writing and editing of the manuscript. All authors and participants reviewed the paper and approved the final manuscript.

## Supporting information

Supplementary Material
