# Supplementary material for: Survival trends and prognostic factors in patients with solitary plasmacytoma of bone: A population‐based study
Source: Cancer Med. 2020 Nov 3;10(2):462–70. doi: 10.1002/cam4.3533 (PMC7877371; doi:10.1002/cam4.3533)
Supplement: Supplementary file 1 — Supplementary Material [file CAM4-10-462-s001.docx]

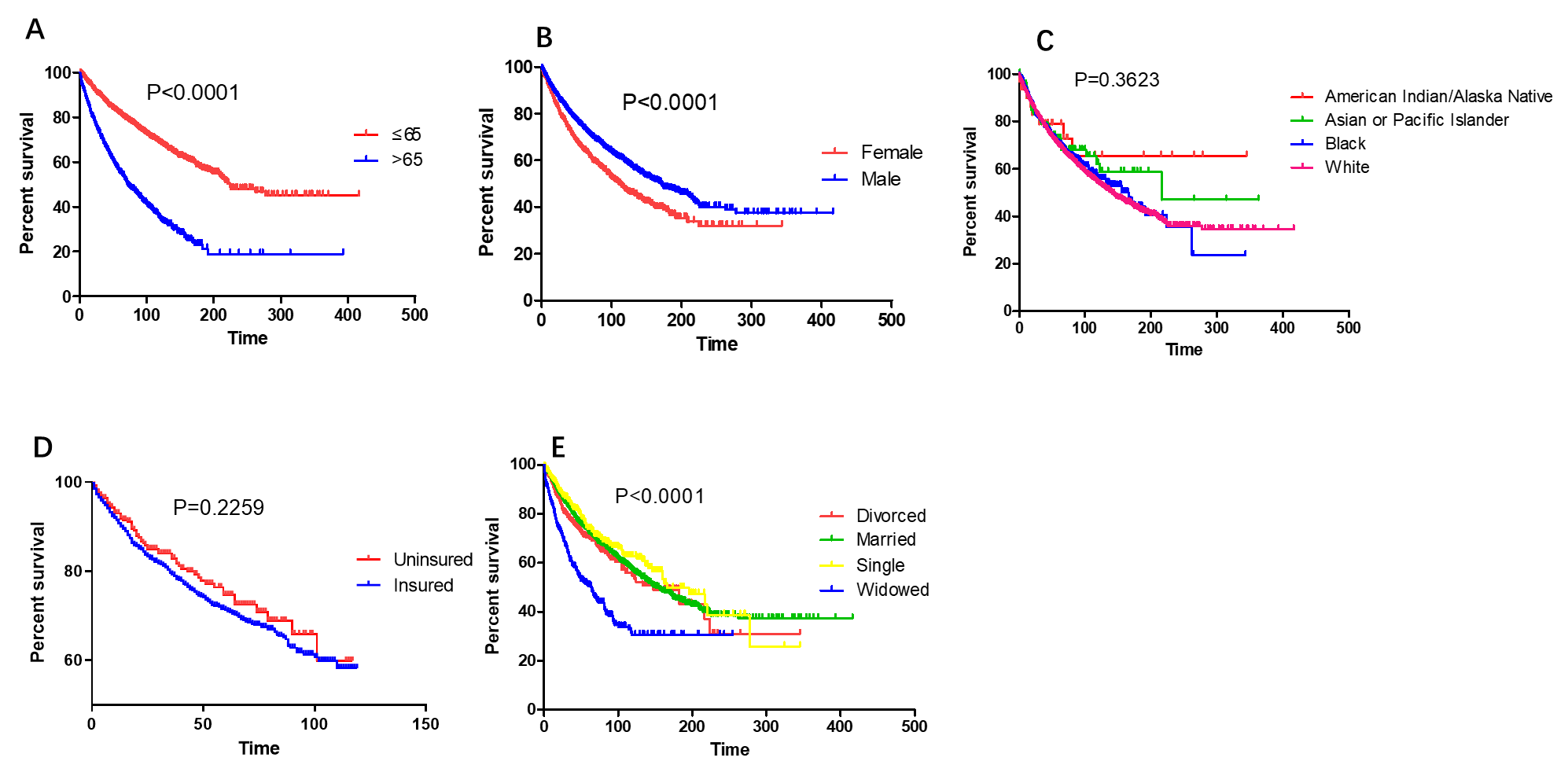


**Supplemental Figure 1**: Myeloma-specific survival of patients with solitary plasmacytoma of bone stratified by age (A), sex (B), race (C), insurance (D), and marital status (E).


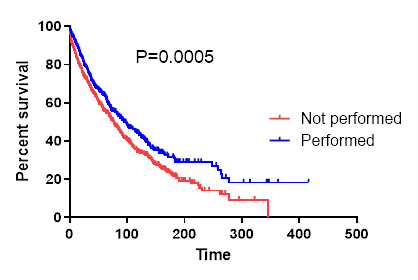


**Supplemental Figure 2**: Survival of patients with solitary plasmacytoma of bone in vertebral column stratified by surgery performed or not.
